# Comparative environmental RNA and DNA metabarcoding analysis of river algae and arthropods for ecological surveys and water quality assessment

**DOI:** 10.1038/s41598-022-23888-1

**Published:** 2022-11-18

**Authors:** Kaede Miyata, Yasuaki Inoue, Yuto Amano, Tohru Nishioka, Tomohisa Nagaike, Takamitsu Kawaguchi, Osamu Morita, Masayuki Yamane, Hiroshi Honda

**Affiliations:** 1 R&D Safety Science Research, Kao Corporation, 2606 Akabane, Ichikai-Machi, Haga-Gun, Tochigi, 321-3497 Japan; 2 Bioindicator Co., Ltd., 18 Iwato-Cho, Shinjuku-Ku, Tokyo, 162-0832 Japan

**Keywords:** Ecosystem ecology, Ecosystem ecology

## Abstract

Environmental DNA (eDNA) metabarcoding is widely used for species analysis, while the use of environmental RNA (eRNA) metabarcoding is more limited. We conducted comparative eDNA/eRNA metabarcoding of the algae and arthropods (aquatic insects) in water samples from Naka River, Japan, to evaluate their potential for biological monitoring and water quality assessment. Both methods detected various algae and arthropod species; however, their compositions were remarkably different from those in traditional field surveys (TFSs), indicating low sensitivity. For algae, the species composition derived from eDNA and eRNA metabarcoding was equivalent. While TFSs focus on attached algae, metabarcoding analysis theoretically detects both planktonic and attached algae. A recently expanded genomic database for aquatic insects significantly contributed to the sensitivity and positive predictivity for arthropods. While the sensitivity of eRNA was lower than that of eDNA, the positive predictivity of eRNA was higher. The eRNA of terrestrial arthropods indicated extremely high or low read numbers when compared with eDNA, suggesting that eRNA could be an effective indicator of false positives. Arthropod and algae eDNA/eRNA metabarcoding analysis enabled water quality estimates from TFSs. The eRNA of algae and arthropods could thus be used to evaluate biodiversity and water quality and provide insights from ecological surveys.

## Introduction

Ecosystem conservation and resource management are crucial global issues addressing sustainable development goals (SDGs)^1^. Hence, ecological surveys are becoming important^2^ for evaluating biodiversity and the spatiotemporal distribution of biological resources. In this context, molecular ecological surveys using environmental DNA (eDNA) metabarcoding, which analyzes DNA released by macro-organisms in aquatic environments, have been touted as a novel alternative to traditional ecological surveys (TFSs) that have low reproducibility and are invasive. Moreover, morphological evaluation using TFSs is limited for species identification^3^. Particularly, the identification of algal, arthropod and aquatic insects in rivers, is hampered by morphological similarities. Furthermore, to conserve genetic diversity, it is necessary to monitor cryptic species that are morphologically inseparable^4^. Therefore, eDNA metabarcoding, which does not require expertise to identify species and is minimally invasive, may contribute to improve the weakness of TFSs^5–7^.

The ability of eDNA metabarcoding analysis to accurately detect many species would be useful to investigate water pollution. Databases of fish fauna monitored using eDNA have been launched such as the All Nippon eDNA Monitoring Network (ANEMONE; https://db.anemone.bio/). Widely implemented molecular ecological surveys and accurate ecological survey results of a vast range of species at numerous locations and time points would help to clarify relationships between ecosystems and environmental factors including water quality, and to apply more effective measures for ecosystem conservation. Yang et al.^8^ used eDNA metabarcoding to analyze microorganisms for community assessment of environmental stressors in a microcosm study. DiBattista et al.^9^ indicated that eDNA could act as a biodiversity barometer in response to anthropogenic pressures in subtropical coral reefs. Molecular biodiversity monitoring using eDNA would be effective for ecosystem management from a water quality perspective because metabarcoding provides a more holistic view of water quality impacts than a few indicator taxa. Thus, species that can be analyzed by eDNA metabarcoding need to be strategically expanded to improve water quality assessment.

Arthropods and algae are promising target taxa of eDNA metabarcoding to evaluate water quality^10–12^. Many indices such as Ephemeroptera, Plecoptera, and Trichoptera (EPT) (%) and average tolerance scores of all macroinvertebrate families found at sites (ASPT), and saprobic scores of diatoms have been developed based on information about the biota of arthropods and algae. Since these indices are based enrichment and not abundance, eDNA metabarcoding would be suitable for evaluating biota. If sufficient biota can be detected by eDNA, it should be possible to estimate the indices obtained from the TFS and assess water quality. Moreover, arthropods and algae have also been used in ecotoxicological evaluations of chemical substances that are important pollutants of aquatic environments^13^ and the application of eDNA in ecotoxicological assessments of such environments has been discussed^14,15^. Thus, monitoring algae, and arthropods in the aquatic environments is also important from both water quality and ecotoxicological perspectives. However, eDNA in aquatic algae and arthropods have not been thoroughly investigated in freshwater areas compared with fish. Moreover, sequencing the mitochondrial genomes of arthropods is rapidly progressing in Japan (Data availability). Thus, sequence data might improve the performance of eDNA metabarcoding.

There are two desirable traits of environmental sensors that contribute to their applicability in ecosystem monitoring; 1) to detect changes in biota, which can be sensitive to environmental variables, with high spatiotemporal resolution; and 2) to detect a wide range of species with high reliability. Since eDNA is stable and can persist in the environment for a long period, the occurrence of false positives was recognized as a major issue^16,17^. Persistence of eDNA might hinder detection of anthropologic effects on the environment. This is where environmental RNA (eRNA) has been attracting attention in recent years^18–20^. Qian et al.^21^ investigated the temperature effects on the stability of *F. chinensis* eDNA/eRNA and found that eRNA showed a faster degradation if temperature was increased, and the stability was maintained across the range of temperature. Furthermore, Kagzi et al.^21^ reported eRNA of *Daphnia pulex* degrades more rapidly than eDNA across a broad range of pH condition and Jo et al.^22^ reported that warm temperature and alkaline conditions accelerate the degradation rate of *Danio rario* eRNA. The degradation rate of eRNA would vary according to species, genes, environmental conditions, and analytical method. Current literature suggests the potential superiority of eRNA over eDNA for ecological surveys with low false-positive rate. Miyata et al.^23^ demonstrated that fish eRNA was abundant in rivers and that eRNA metabarcoding analysis was useful for precise ecological surveys with high positive predictivity. Subsequently, Littelefair et al.^24^ reported a higher true positive rate of eRNA in comparison to monitoring freshwater habitats using eDNA and eRNA in a lake. In addition, Broman et al.^25^ reported the utility of shotgun sequencing using the eRNA of microeukaryote groups, including Arthropoda and Bacillariophyta, to investigate biodiversity. However, the abundance and usefulness of eRNA to discern other algae species and macrofauna (e.g., aquatic insects) in rivers remain unclear. Moreover, since fish eRNA contributed to classifying false positives between saltwater and brackish water fish^23^, algae and arthropod eRNA may be able to distinguish habitat properties (e.g. terrestrial and marine organisms) relating to false positives.

Thus, in the present study, the following hypotheses were investigated: (1) sufficient amount of eRNA is available for metabarcoding analysis of river arthropods and algae; (2) recently-obtained arthropod sequences contribute to the refinement of detectability; (3) eRNA indicates higher positive predictivity and discriminates potential false positives; (4) water quality indices derived from eDNA and eRNA are in agreement with those derived from TFS, and eRNA is a superior indicator for water quality assessment; and (5) eDNA/eRNA metabarcoding is a promising alternative method for traditional ecological surveys. To this end, we conducted comparative eDNA/eRNA metabarcoding analyses using next-generation sequencing in Naka River to confirm the abundance of algae and arthropod eRNA, and the survey results were compared with TFS results, which were conducted simultaneously with eDNA/eRNA analysis for the performance evaluation of an ecological survey. The Naka River is one of the representative large rivers in Japan, flowing through the Nikko National Park in the upper reaches and the Yamizo and Naka River Prefectural Natural Parks in the middle reaches. Hence, the surrounding environment retains much of its natural scenery and is home to a wide variety of organisms. Over the years, fish and arthropod surveys have been performed regularly, and fish eDNA/eRNA metabarcoding analysis has also been conducted, which has the advantage of facilitating the interpretation of environmental nucleic acid results. In the analysis, to elucidate the differences in species detected using eDNA and eRNA, terrestrial arthropods and brackish and saltwater algae were defined as potential false positives that might not inhabit the sampling points. Moreover, we examined the effects of using a recently expanded genomic database on species detection performance in arthropods and compared water quality indices that were derived from eDNA/eRNA and TFS. This study provides multifaceted information on arthropods and algae eRNA in rivers.

## Methods

### eDNA and eRNA samples

Water samples were collected and eDNA/eRNA extracted, as previously described by Miyata et al.^23^. The water samples (3.0 L, n = 22: 1 sample/site/day × 2 sites × 11 days) were collected, using buckets, twice or thrice a month during autumn and winter (2018–2019) at Nakagawa-oohashi (N36°32′55″, E140°19′34″ E) and Shin-nakabashi (N36°45′26″, E140°08′30″ E) on the Naka River in Japan. The Naka River is a large river with a basin area of approximately 3,000 km^2^. The upstream point (Shin-nakabashi) is a flat stream with rapids, and the downstream point (Nakagawa-oohashi) is also a flat stream. Water was collected with buckets over several times and places, including rapids where aquatic arthropods were abundant, taking care to avoid contamination with mud or other substances that may cause PCR inhibition. Water was collected universally from shallows at a depth of approximately 30 cm near the shore to a flowing area at a depth of 1 m or more in the middle of the river, and the water was mixed evenly as samples. When the depth was more than 1 m, water was collected with the bucket, which was immersed under water. Then, Sterivex™ filter units were used to filter the water samples (nominal pore size, 0.45 μm; Millipore, Billerica, MA, USA), which were subsequently filled with ice-cold RNAprotect Tissue Reagent (Qiagen, Hilden, Germany). Purified water was filtered and used as the negative control. The negative controls were tested for evidence of contamination during the experimental procedures. Total eDNA was extracted using an extraction buffer with RNase A, proteinase K, and 15% polyvinylpolypyrrolidone, and buffer AL from a DNeasy blood and tissue kit (Qiagen, Hilden, Germany). Next, it was purified according to Miya et al.^26^ using an MPure bacterial DNA extraction kit (MP Biomedicals, Santa Ana, CA, USA) and AMPure XP (Beckman Coulter, Brea, CA, USA). Total eRNA was extracted using a ChargeSwitch total RNA cell kit (Thermo Fisher Scientific, Waltham, MA, USA) with DNase treatment. Sample preparation was as follows. Sterivex™ filter units were filled with 1,000 μL of the prepared Lysis Mix and incubate at 60 °C for 1 h. After incubation, the lysate was collected by centrifugation and transferred into a 1.5 mL microcentrifuge tube. Then, the lysate was mixed briefly by vortexing and cooled the samples for 1 min on ice. From then on, we followed the manufacturer's protocol. Then, cDNA was synthesized using a PrimeScript II 1st strand cDNA synthesis kit (Takara Bio Inc., Shiga, Japan), following the manufacturer's protocol. The negative controls of arthropod eDNA/eRNA and algal eRNA (except for algal eDNA) were not analyzed further because no target DNA or cDNA was amplified. The algal eDNA negative controls were used in the next step.

### Amplicon library preparation and next-generation sequencing

The amplicon library was prepared, and MiSeq sequencing was conducted as previously described by Miyata et al.^23^. Briefly, amplicon libraries of partial 16S rRNA and *psbA* genes were generated by PCR amplification using primers targeting the mitochondrial 16SrRNA of arthropods (gInsect) and the *psbA* gene^27^ (Table 1). The first and second PCR (and purification of amplicons) were performed according to Miyata et al.^23^. However, for *psbA*, we modified the conditions of the first PCR. *psbA* underwent initial denaturation at 94 °C for 2 min, followed by 35 cycles of denaturation at 94 °C for 30 s, and annealing at 52 °C for 30 s. Then, it was extended at 72 °C for 30 s with a final extension at 72 °C for 5 min. The first PCR amplification was replicated four times, and the second PCR used adaptor sequences and 8 bp index sequences.

**Table 1:** PCR primers used in the present study.

Primer name	Sequence (5′ → 3′)
gInsect F	ACACTCTTTCCCTACACGACGCTCTTCCGATCTNNNNNNGATAGAAACCAACCTGGCT
gInsect R	GTGACTGGAGTTCAGACGTGTGCTCTTCCGATCTNNNNNNGACGAGAAGACCCTATA
psbA_597f	ACACTCTTTCCCTACACGACGCTCTTCCGATCTTNCAYTTCTAYCCNVTHTGGGA
psbA_927r	GTGACTGGAGTTCAGACGTGTGCTCTTCCGATCTRNCATGTGGAATGGGTGCAT

### Bioinformatics analysis of high-throughput sequencing data

Bioinformatics analysis was conducted according to Miyata et al.^23^. First, multiple FASTQ files, matched against the beginning of the specified index reads, were extracted using the fastq_barcode_spliltter tool in the program FASTX-Toolkit (ver. 0.0.14). Amplicon sequence variants (ASVs) were produced using DADA2^28^. The primer sequences in *psbA* were trimmed 50 bp from the 3-terminal end. Next, chimeras and noise sequences were removed using Qiime 2 plugin wraps DADA2 with default settings^29^ (ver. 2020.8). In addition, a sequence table was created as a matrix in which each row corresponds to a processed sample and each column indicates a non-chimeric inferred sample sequence. All processed sequences for algae and arthropods were subjected to BLASTN search (ver. 2.9.0) against the NCBI nonredundant nucleotide sequence database, and those for arthropods were also subjected to Mitochondrial Genome Database of Benthos and Insect (for Environmental Research) in Japan (MBIJ) developed independently by the researchers of the Bioengineering Lab. Co., Ltd. et al. (see Data availability section). The list of species included in MBIJ is provided in Supplementary Table S1. The BLAST hit with a sequence identity of ≥ 97% and an alignment length of ≥ 100 bp was applied to species assignments of each representative sequence (Supplementary Table S2). The Qzv file was exported to another file type.

### Traditional field survey (TFS)

Arthropod TFSs were conducted using a method adapted from the Manual of the National Census of River Environments^30^. Samples were collected via kick-sampling or wash-sampling using dip nets in each identified instream habitat (e.g., riffles, pools) and a Surber sampler measuring 25 cm × 25 cm (0.5 mm mesh) during autumn and winter (January and November 2019). Sampling was conducted once per season, with its date coinciding with the water sample collection dates for eDNA/eRNA analysis. Samples were collected at three points in riffles at each research site/season using a Surber sampler.

The TFSs for algae were conducted with a focus on epilithic (attached) algae simultaneously with arthropod TFSs. Three cobbles were collected from riffles at each research site/season, and algae were scraped from 5 × 5 cm sampling areas on each cobble. Samples were also collected from each identified instream habitat by scraping the cobbles (the sampling area was not fixed).

### Performance evaluation of eDNA/eRNA metabarcoding analyses in ecological surveys

To examine the performance of the eDNA/eRNA metabarcoding analysis, their sensitivity and positive predictivity were calculated as follows:

$${\text{Sensitivity}} = ({\text{number of true positives}})/({\text{number of positives in TFS}})$$

$${\text{Positive predictivity}} = ({\text{number of true positives}})/({\text{number of positives in metabarcoding analysis}})$$

In this analysis, true positives were defined as “identified species using metabarcoding analysis of the observed species in TFS.” Specificity and negative predictivity were not evaluated as there were no true negatives in the TFS. Sensitivity indicates the extent to which eDNA and eRNA metabarcoding can detect species found in the TFS. Positive predictivity indicates the extent to which the species detected by eDNA eRNA metabarcoding truly exist. Similar indicators were used to verify the accuracy of the analysis^31–33^.

The results of even highly sensitive methods cannot be trusted, if they generate more false positives than true positives. When the prevalence (total detected species in TFS/total species in TFS and metabarcoding analysis) of the target species is low or the target species is likely to be absent or at low abundance, positive predictivity would be more important than sensitivity^32^. Moreover, recent studies of eRNA^23,24^ have applied the false discovery rate (1-false positive rate) or positive predictivity in addition to the normal detection rate (sensitivity, true positive rate).

The average total read numbers at the two sampling points were used to compare the merged TFS data (Nakagawa-oohashi and Shin-nakabashi). Species with read numbers < 10 bp for both eDNA and eRNA were excluded. In the arthropod analysis, sensitivity and positive predictivity were evaluated using two types of TFS data: 1) quantitative data only and 2) quantitative and qualitative data (entire data). The performance was also evaluated for each taxon, focusing on Ephemeroptera, Plecoptera, Trichoptera, Odonata, and Diptera (EPTOD). Furthermore, we confirmed the performance of metabarcoding analysis for arthropod ecological surveys using the TFS database of the Naka River^34^. However, since data about the 16S rRNA sequence have recently expanded, we compared performance using current and expanded databases (species included in current database plus MBIJ). Finally, we created Venn diagrams to compare the number of species detected using eDNA, eRNA, and TFS under optimal analytical conditions (TFS, quantitative data; metabarcoding, expanded database). Arthropods’ species that were detectable and undetectable in the eDNA/eRNA metabarcoding analysis were analyzed in terms of sequence registration in the database and the ratio of each detected taxon. In addition, the top 30 arthropod species in terms of abundance detected by TFS and read numbers after metabarcoding analysis were also compared using a heatmap. We then analyzed the status of sequence registration in the database.

### Usefulness of arthropod eDNA/eRNA metabarcoding analysis for water quality evaluation.

We evaluated the usefulness of eDNA/eRNA metabarcoding analysis for deriving water quality indices. The eDNA/eRNA metabarcoding analysis and arthropod TFS data were used to calculate Ephemeroptera, Plecoptera, and Trichoptera (EPT) (%) and the average tolerance scores of all macroinvertebrate families found at the site (ASPT), which were utilized to evaluate water quality. The ASPT was calculated according to the manual for water quality assessment for aquatic organisms in Japan^10^. Then, we compared the EPT (%) and ASPT scores. Higher EPT (%) and ASPT scores indicate cleaner water in the water quality evaluation.

The eDNA/eRNA metabarcoding analysis and algae TFS data were used to evaluate water quality by the saprobic score of diatoms. The saprobic score was calculated according to Kobayashi & Mayama^11,12^. A higher saprobic score indicates cleaner water in water quality evaluation.

### Differences in eDNA/eRNA metabarcoding analyses for classifying false positives

To discuss the capacity to distinguish living biotic assemblages from dead biotic assemblages, terrestrial arthropods and brackish and saltwater algae were defined as potential false positives that were not likely to inhabit the sampling points. Moreover, the features of attached/planktonic algae were analyzed but not defined as false positives as planktonic algae are not usually evaluated in TFSs. The habitats were examined using information from the MLIT-RE database^34^, scatter plots between eDNA and eRNA were illustrated for all species detected, and the distributions were compared using box plots.

### Statistical analyses

One-way ANOVA, followed by the Tukey–Kramer test, was employed to analyze statistical differences in sensitivity and positive predictivity for multiple comparisons under different analysis conditions for arthropods. The sensitivity and positive predictivity for algae were analyzed using Student’s *t*-test. EPT (%), ASPT, saprobic score, and read numbers were evaluated by using a paired *t*-test. Mann–Whitney *U* test was conducted for analyzing differences in read numbers between aquatic- and terrestrial- arthropods. All statistical analyses, except for the Tukey–Kramer test, were performed using Origin 2019b (OriginLab, Northampton, MA, USA). Tukey–Kramer test was performed using the multcomp package in R software. Statistical significance was set at P < 0.05.

## Results

### Performance of eDNA/eRNA metabarcoding analysis for ecological surveys

**Figure 1:**
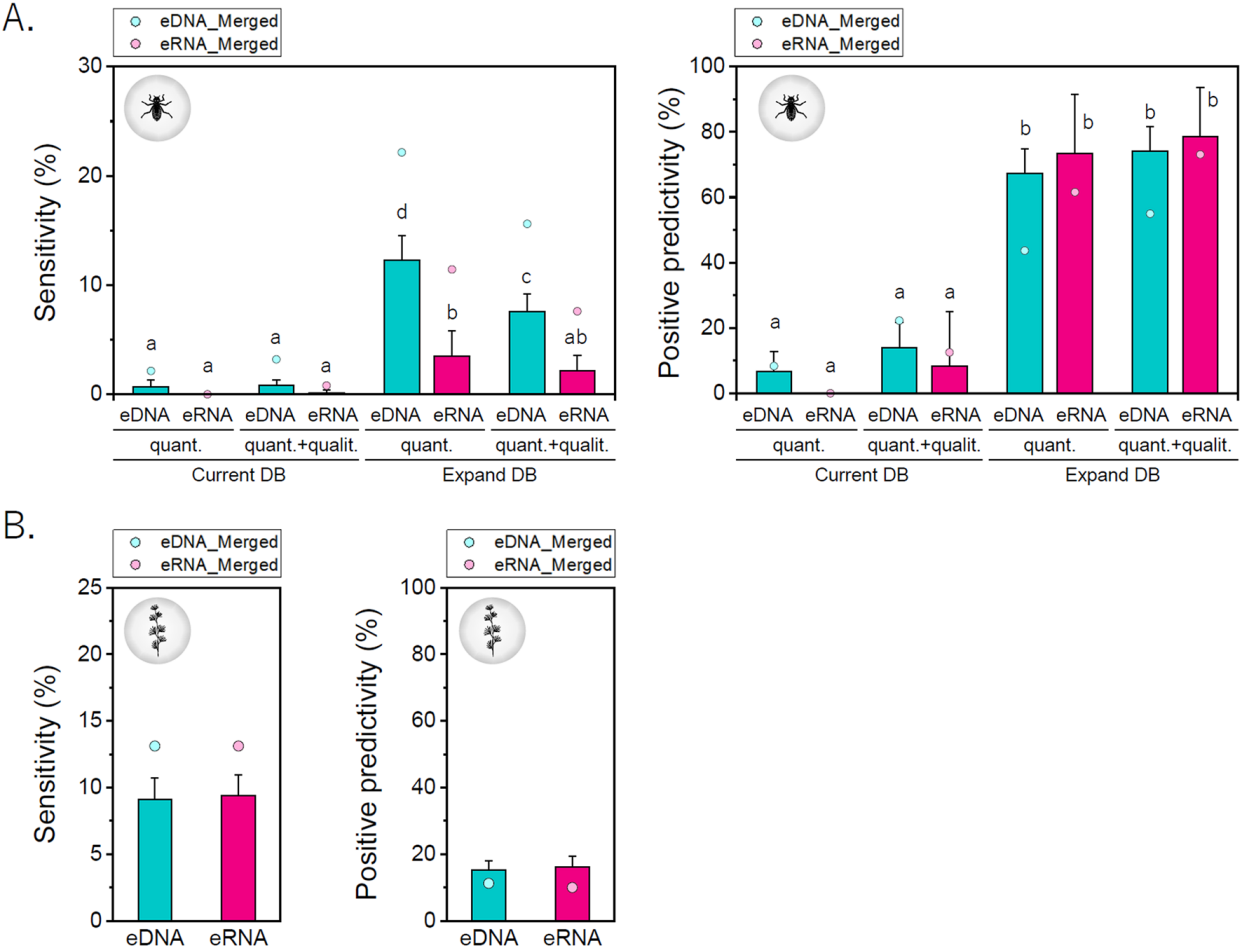
Performance of eDNA/eRNA metabarcoding analysis in ecological surveys of arthropods and algae. Sensitivity and positive predictivity of metabarcoding analysis for (**A**) arthropods and (**B**) algae. eDNA/eRNA: eDNA/eRNA metabarcoding analysis, quant.: quantitative analysis, qualit.: qualitative analysis. Dots indicate the performance when individual data are merged. The values represent the mean + standard deviation (SD). Different letters indicate significant differences (Tukey–Kramer test). The values represent the mean + standard deviation (SD). Arcsine transformation did not change the letters.

Sensitivity and positive predictivities were compared across different experimental conditions (Fig. 1). The sensitivity and positive predictivity of the eDNA/eRNA metabarcoding analysis in arthropods both drastically improved when an expanded database was used (merged data; current database: 0–3.2%/0–22.2%, expanded database: 7.6–22.1%/43.7–73.1% (sensitivity/positive predictivity)) (Fig. 1A, Supplementary Table S3). The sequences in the expanded database improved the performance of the metabarcoding analysis. Furthermore, when only quantitative data were used as TFS data, there were maximum sensitivity values for the eDNA and eRNA. However, positive predictivities using quantitative analysis slightly decreased when compared with those using quantitative and qualitative data (no significant differences were found). Similar results were confirmed for the metabarcoding of arthropod ecological surveys using the MLIT database (Supplementary Fig. S1). Thus, in this study, we mainly analyzed the conditions with high sensitivity (using quantitative TFS data obtained in the present study and an expanded database of metabarcoding analysis) for arthropods.

When comparing eDNA and eRNA, the sensitivity of eDNA was significantly higher than that of eRNA in the expanded database. In contrast, the positive predictivity of eRNA was higher than that of eDNA, although no significant difference was found.

When individual datasets were merged, sensitivity increased, and positive predictivity decreased. The order of the performance of eDNA and eRNA did not change. However, the percentage increase in sensitivity with the merged data was higher for eRNA (eDNA: 12.3–22.1% [× 1.79], eRNA: 3.5–11.4% [× 3.25]). In contrast, the percentage decrease in positive predictivity was higher for eDNA (eDNA: 67.4–43.7% [× 0.83], eRNA: 73.4–61.5% [× 0.64]). This indicates that eRNA can easily increase sensitivity, and eDNA may be prone to generating false positives on using merged data.

The performance of each taxon was also evaluated, focusing on the EPTOD (*Table **2*). Plecoptera was not detected in eDNA or eRNA metabarcoding. In terms of sensitivity, Ephemeroptera had the highest average score for eDNA and Diptera had the greatest score for eRNA. Odonata had the highest merged score for both eDNA and eRNA. In terms of positive predictivity, the average and merged scores were the highest for Ephemeroptera in eDNA and Trichoptera in eRNA.

**Table 2:** Sensitivity and positive predictivity of metabarcoding analysis in each class.

	eDNA			eRNA		
	Mean	SD	Merge	Mean	SD	Merge
**Sensitivity**						
Ephemeroptera	30.0	6.8	53.3	4.1	5.6	26.7
Plecoptera	0.0	0.0	0.0	0.0	0.0	0.0
Trichoptera	10.4	4.7	21.4	1.6	3.0	7.1
Odonata	27.3	24.9	100.0	5.6	15.7	50.0
Diptera	20.9	3.6	35.0	15.0	5.8	20.0
**Positive predictivity**						
Ephemeroptera	86.9	6.1	84.2	38.6	28.2	80.0
Plecoptera	0.0	0.0	0.0	0.0	0.0	0.0
Trichoptera	56.1	21.6	42.9	100.0	0.0	100.0
Odonata	26.7	22.6	25.0	16.7	23.6	25.0
Diptera	70.3	12.0	50.0	96.3	10.5	80.0

In algae, the sensitivity was mostly equivalent. The average score was slightly higher for eRNA (eDNA 9.1%, eRNA 9.4%), and the merged score was equal (no significant difference). Positive predictivities were also similar. The average score was higher for eRNA, whereas the merged score was higher for eDNA. However, no significant differences were found (Fig. 1B).

### Differences in the species detected using eDNA/eRNA vs. TFS analyses

**Figure 2:**
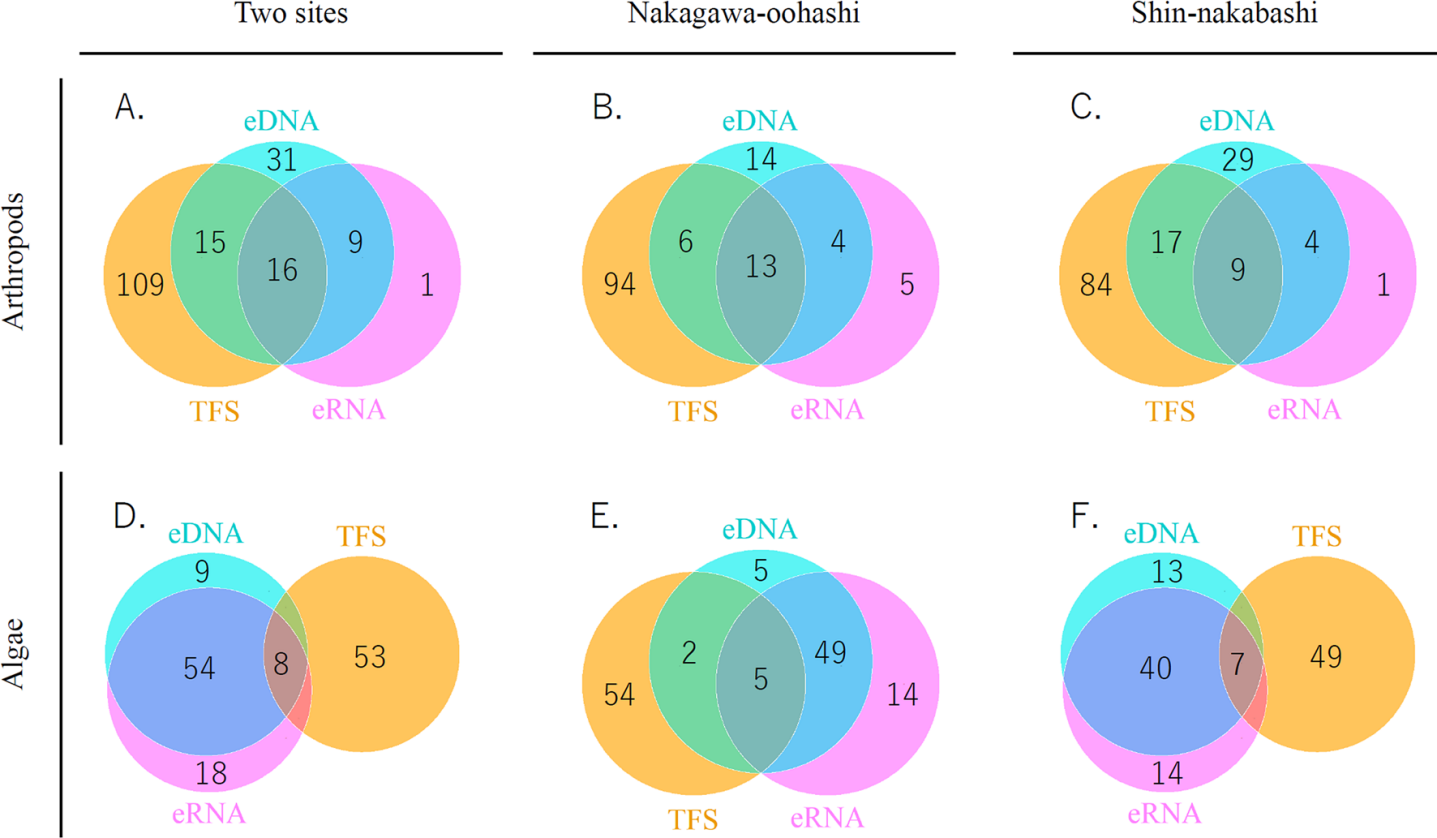
Comparison of the species detected using the eDNA/eRNA metabarcoding analyses and traditional field surveys (TFSs). (**A–F**) Comparison of the species detected using eDNA/eRNA metabarcoding and TFS. Venn diagrams were illustrated for multiple analytical conditions in a matrix (species vs. sites). For example, (**A**) indicates a result using two-site merged data in arthropods, and (**E**) uses data only from Nakagawa-oohashi for algae.

Venn diagrams illustrate the differences in species detected between the eDNA/eRNA metabarcoding analysis and TFS under multiple analytical conditions (Fig. 2). For the arthropods, eDNA and eRNA analyses detected 71 and 26 species, respectively. The TFS detected 140 species at two sampling points (Nakagawa-oohashi and Shin-nakabashi). The lower species detection rate with eRNA was probably responsible for the low sensitivity and high standard deviation of positive predictivity in eRNA (Fig. 1A). Although 25 species were commonly detected in the eDNA/eRNA analyses, only 16 species were common in the eDNA/eRNA analyses and TFS. The sensitivity of eRNA detection (11.4%) was lower than that of eDNA detection (22.1%). The positive predictivity of eRNA (61.5%) was higher than that of eDNA (43.7%).

For algae, eDNA and eRNA analyses detected 71 and 80 species, respectively, equivalent to the number detected with the TFS (61). However, only 8 species were common with the TFS results. Conversely, almost all species (62) were common in eDNA/eRNA analyses. The sensitivity and positive predictivity of eDNA/eRNA were equivalent (sensitivity, 13.1%; positive predictivity, 10–11.3%). The TFS focuses on algae attached to stones in rivers, whereas eDNA/eRNA was collected from surface water. Hence, eDNA/eRNA analyses could detect species that were identified by TFS when using bottom water. There were no issues with the quality and interpretation of the results since only one species, *Musa* sp., a terrestrial plant, was detected in algal eDNA negative controls, and it was not in other samples.

**Figure 3:**
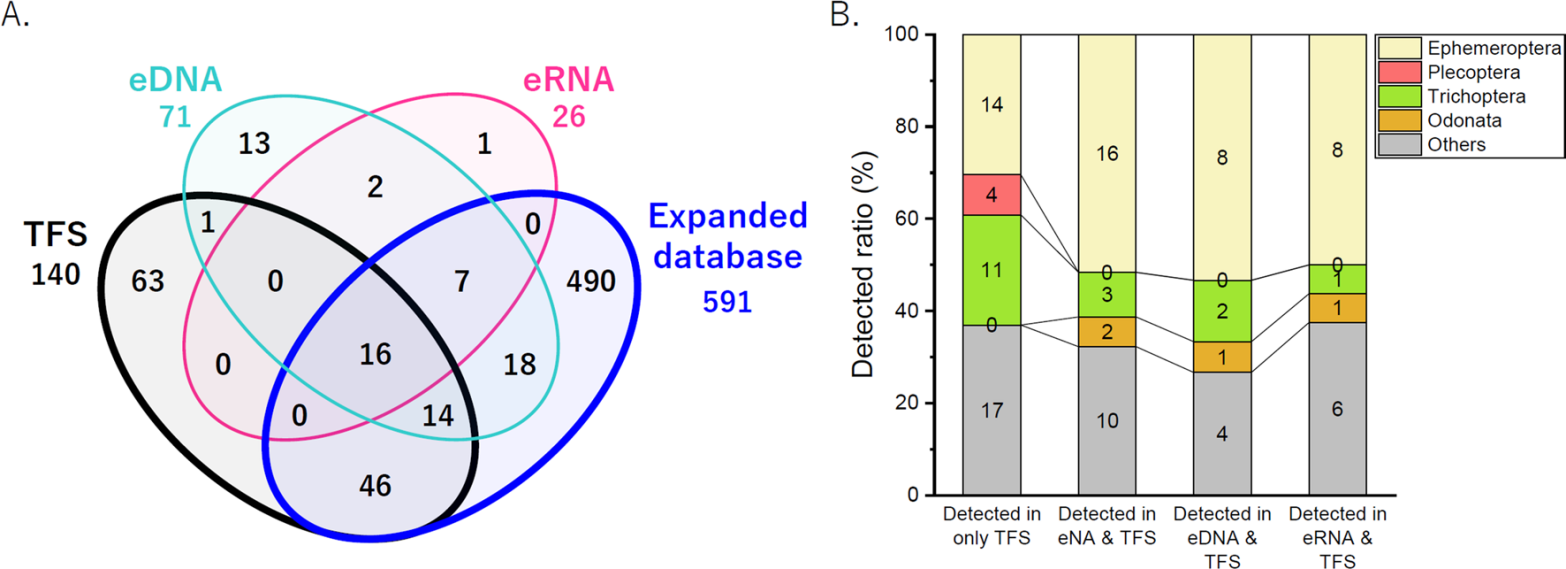
Number of arthropod species detected in the traditional field survey and metabarcoding analysis and their sequence data availability in an expanded database. (**A**) Comparison of the arthropod species detected using eDNA/eRNA metabarcoding and traditional field surveys (TFSs), and the existence of recently registered species in the expanded database. (**B**) Comparison of the ratios between species detected in environmental nucleic acids (eNA [eDNA and eRNA]) and TFS and species detected in only TFS.

A total of 109 species (63 + 46 species in Fig. 3A) were not detected in the metabarcoding analysis, but were detected in the TFS. Moreover, 46 of the 109 species have already been registered in an expanded database. Then, we compared the composition of the species detected with the eDNA/eRNA metabarcoding analysis and those only detected in the TFS (Fig. 3B). The results showed that the EPTO ratio was similar (approximately 60–70%). However, Plecoptera was not detected in eDNA/eRNA metabarcoding, whereas all Odonata species could be detected in that analysis.

### Comparison of the read numbers from the metabarcoding analysis and TFS abundance

**Figure 4:**
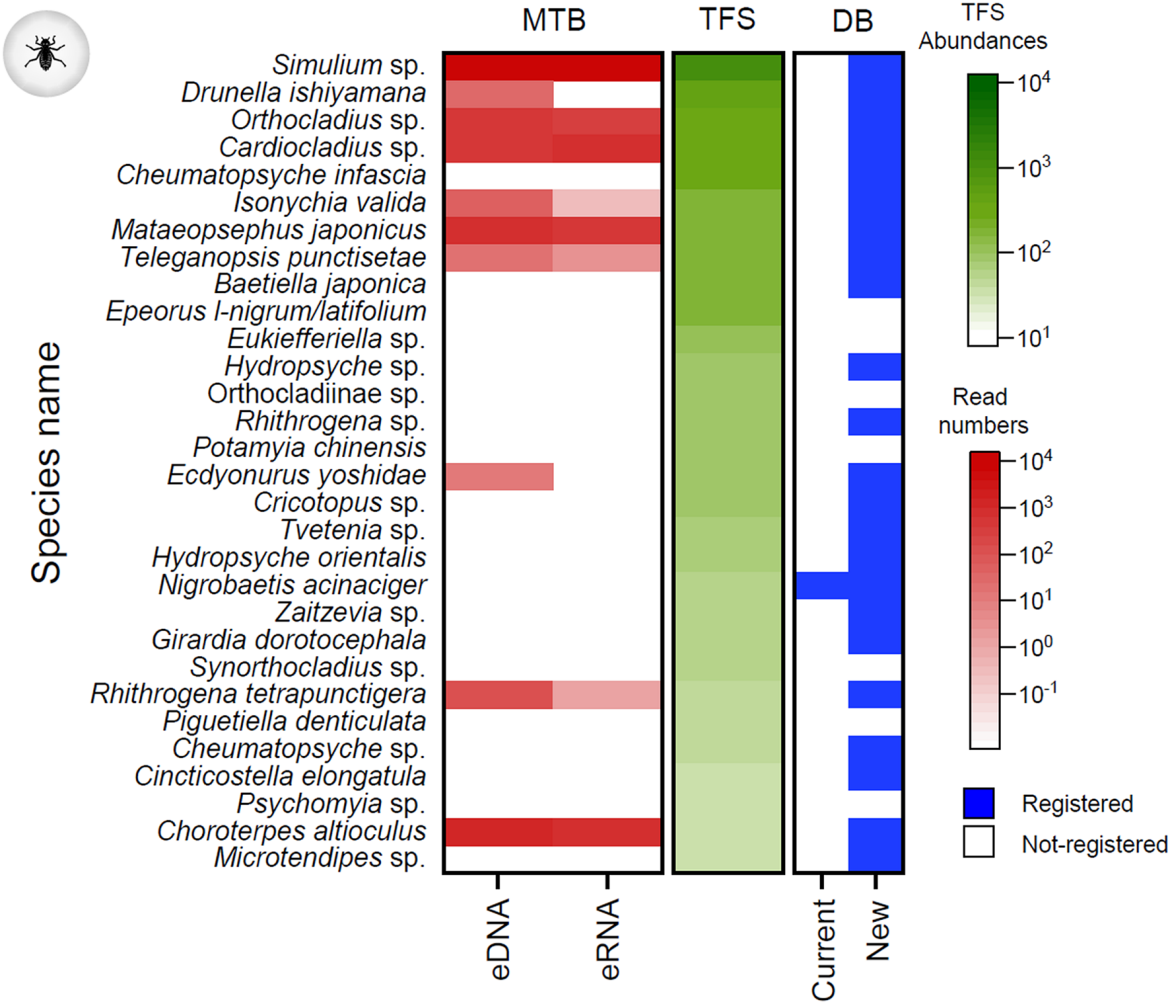
The 30 arthropod species with the greatest abundance detected in traditional field surveys and their read numbers in metabarcoding analysis. MTB: metabarcoding analysis, TFS: traditional field survey, DB: database. Current: current database, New: recently registered species in the expanded database. Read numbers in eDNA/eRNA metabarcoding analysis and abundances in TFS were illustrated.

The abundance of the top 30 arthropod species in the TFS and their read numbers in the metabarcoding analysis were illustrated using a heatmap (Fig. 4). No species were detected in the eRNA metabarcoding only, and the eDNA identified all species detected in the eRNA. For several species (e.g., *Drunella ishiyamana* and *Isonychia valida*), the eDNA read numbers were remarkably higher than the eRNA read numbers. Of the top 30 species in TFS, 10 were found in eDNA metabarcoding. Of these 10 species, 7 were found in eDNA and 6 were detected in eRNA. Of the 20 species not found in eDNA/eRNA metabarcoding, 10 have been already registered in the expanded database. Further investigation is needed to determine why each species was not detected by metabarcoding, as PCR bias should be considered when comparing metabarcoding read counts and TFS abundance. However, since the primer sequences were the same for eDNA and eRNA and the insert sizes were almost identical, comparisons between eDNA and eRNA might have some meaning.

### Comparison of water quality indices in eDNA/eRNA metabarcoding and TFS

To evaluate the usefulness of metabarcoding analysis as a water quality index, the EPT (%) and ASPT scores derived from arthropod TFS and saprobic score derived from algae TFS were compared with those derived from eDNA/eRNA metabarcoding analyses.

**Figure 5:**
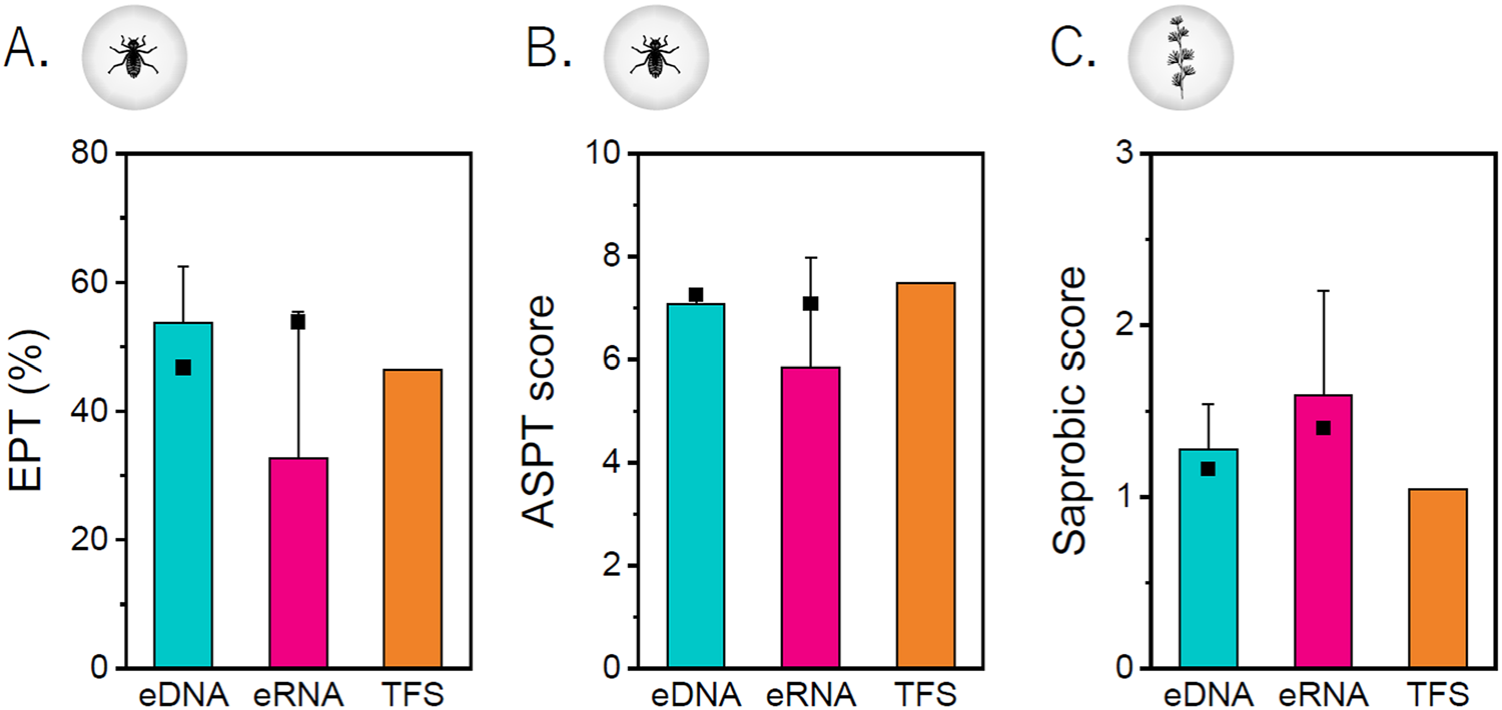
Comparison of water quality indices derived from eDNA/eRNA metabarcoding analysis and TFS. EPT (%): Ephemeroptera, Plecoptera, and Trichoptera (EPT) ratios. ASPT: the average score per taxon. (**A**) Comparison of EPT (%) between eDNA/eRNA metabarcoding analysis and TFS. (**B**) Comparison of the ASPT scores between the eDNA/eRNA metabarcoding analysis and TFS. (**C**) Comparison of saprobic scores between the eDNA/eRNA metabarcoding analysis and TFS. Square; merged score of eDNA/eRNA.

For EPT (%), the average score derived from the eDNA was slightly higher than that derived from TFS. In contrast, the average score derived from eRNA was lower than that derived from arthropods TFS (Fig. 5A). The average value of EPT (%) derived from the eDNA was significantly higher than that derived from the eRNA (p = 0.03; paired *t*-test). When comparing the merged scores, eRNA had the highest score when compared with eDNA and arthropods TFS. The average ASPT score derived from arthropods TFS was higher than that from the eDNA and eRNA. The average value of ASPT derived from the eDNA was significantly higher than that derived from the eRNA (p = 0.01; paired *t*-test). When comparing the merged scores, the ASPT scores derived from the eDNA, eRNA, and arthropods TFS were equivalent (Fig. 5B). These data suggest that individual surveys using eDNA can allow the stable evaluation of water quality indices derived from the arthropods TFS. However, the individual values of the indices derived from eRNA may be slightly lower than those from arthropods TFS, while merged values are likely to be equivalent to those from the arthropods TFS. For EPT (%) and the ASPT scores, the merged score derived from the eDNA and eRNA indicated the same water quality rank as the score derived from arthropods TFS (30 ≤ EPT: Good water quality; 6.0 ≤ ASPT < 7.5: Good water quality [2nd of 4 levels]).

With regard to the saprobic score, the average score derived from the eDNA and eRNA was slightly higher than that derived from algae TFS (Fig. 5C). The average value of the saprobic score derived from the eDNA was significantly higher than that derived from the eRNA (p = 0.04; paired *t*-test). When comparing the merged scores, eRNA had the highest score when compared with eDNA and algae TFS. These data indicate that the individual values of the indices and merged values derived from eDNA and eRNA may be slightly higher than those from algae TFS. The merged score derived from the eDNA and eRNA indicated the same water quality rank as the score derived from algae TFS (1 ≤ saprobic score < 1.5: Good water quality [1st of 4 levels]).

### Effect of ecological traits related to false-positive generation in eDNA/eRNA metabarcoding analysis

**Figure 6:**
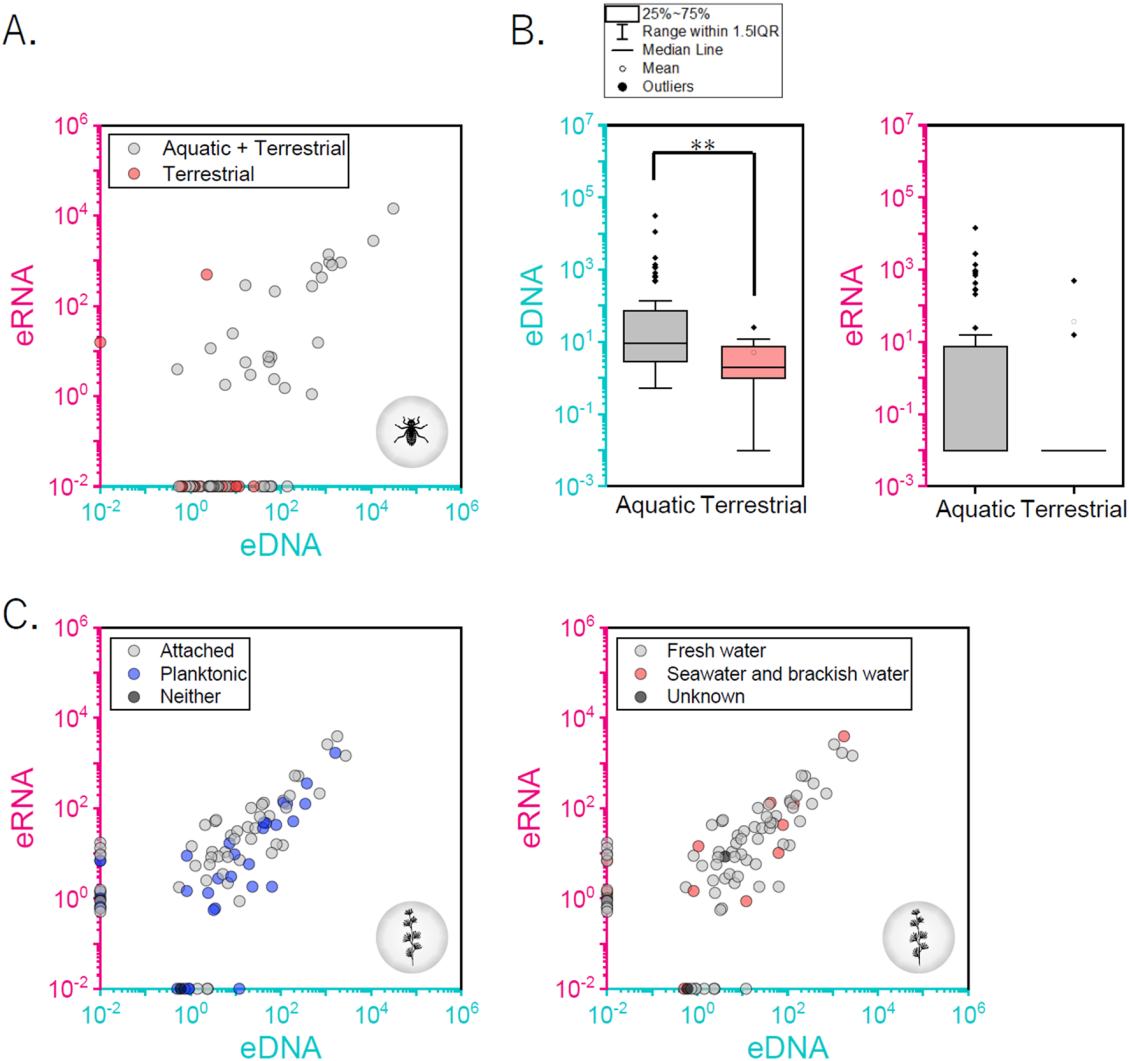
Ecological trait evaluation using eDNA/eRNA metabarcoding analyses to identify false positives. (**A**) Scatter plot between eRNA and eDNA read numbers for arthropods and species' habitat. (**B**) Differences in eDNA/eRNA read numbers between aquatic and terrestrial arthropods. (**C**) Scatter plot between eRNA read numbers and eDNA read numbers in algae and ecological traits related to false positives, (Left) attached or planktonic (Right) fresh water or seawater and brackish water. **p < 0.01.

Some species of arthropods were detected only in the eDNA analysis, and the common functions of these species were explored. Interestingly, terrestrial arthropods were mainly detected in eDNA and not in eRNA. However, two terrestrial species (*Pteronemobius fascipes* and *Nysius plebeius*) had extremely high read numbers in eRNA when compared with those in the eDNA (Fig. 6A). These results suggest that the amount of eRNA in terrestrial arthropods can be extremely high or low. Thus, eRNA could act as an indicator to determine whether a species is actually inhabiting the water environment or if it enters from the outside. Moreover, the eDNA read numbers of aquatic arthropods were significantly higher than those of terrestrial arthropods (Fig. 6B). Hence, the combined analysis of eDNA and eRNA may provide an effective means to detect false positives.

In algal metabarcoding analysis, common ecological traits were not found in species detected only in eDNA or eRNA. Planktonic and attached algae, together with seawater and brackish algae were distributed homogeneously (Fig. 6C).

## Discussion

Our results indicate that the eRNA from a variety of algal and arthropod species is maintained in rivers, which enables metabarcoding analysis. It has been reported that the eRNA of various aquatic species are available in the marine^35–42^. Additionally, sufficient amounts of fish eRNA for ecological survey have been detected in rivers^23^. These results indicate that the eRNA of major trophic species, including herbivores and predators in rivers, is likely maintained in all water environments. This is also consistent with the view that eRNA has a certain level of stability^43–45^ that allows its maintenance in the environment^20^.

The performance of eDNA/RNA metabarcoding in ecological surveys did not achieve sufficient levels for either algae or arthropods when existing databases were used. Sensitivity and positive predictivity for the algae and arthropods were low when compared with those previously reported for fish^23^. For algae, although the detected eDNA species were in good agreement with those of the eRNA when compared with arthropods, unlike the hypothesis, significant differences were not observed in sensitivity and positive predictivity. The lack of differences in algae may be associated with the direct capture of algae in the filter units. As the size of algae exceeded the pore size^46^, our protocol may have captured the eDNA and eRNA of algae, regardless of whether they were dead or alive. Although several studies have reported that eRNA is a sensitive marker of environmental changes^47^, further analysis is required to investigate the differences between eDNA and eRNA metabarcoding. Nonetheless, a novel aspect identified in this study regarding eDNA/eRNA metabarcoding was that both planktonic and attached algae could be detected. The species detected in metabarcoding were similar to those identified using TFSs. However, half of the species identified were planktonic algae, indicating that they were associated with low performance. Metabarcoding analysis, which can be used to analyze planktonic algae, may be useful for investigating a wide range of species. TFSs or metabarcoding analysis using bulk samples, which target only non-moving attached algae, could be useful for investigating their relationship with environmental factors.

The identification of arthropods was extremely low when using the existing sequence database for metabarcoding; however, it immensely improved when the expanded database was used. In particular, positive predictivity showed a maximum value in eRNA (combined data)—as the value was changed from approximately 0% to 80%, which was considered sufficient. This suggests that the sequence registration of organisms found at the site may be an efficient approach to enhance eDNA/eRNA metabarcoding for ecological surveys. Additionally, this may increase positive predictivity for arthropods. High positive predictivity indicates the reliability of positive metabarcoding results, and eRNA metabarcoding might be useful for analyzing the distribution of rare species. To further improve the sensitivity, two approaches should be considered. The first strategy involves the sequencing and registration of species not identified by metabarcoding. However, of the 109 species that were not detected, 46 have been already registered in the expanded database. Thus, sequence registration alone has limitations in improving the sensitivity. Crustacean eDNA is released in small quantities^48^ as their bodies are covered by exoskeletons. Therefore, even arthropods covered with exoskeleton may release a small amount of eDNA and eRNA, reflecting the fact that EPTO taxa are difficult to detect in water samples^49^. Hence, the development of a new approach for collecting eDNA and eRNA is necessary. For example, a bulk analysis may be conceivable, wherein fragments of arthropods may be collected from sediment in rivers, regardless of whether they are alive or dead. Metabarcoding analysis using bulk samples can reportedly improve performance^49^. Second, water samples can be collected from various subsites. TFS qualitative analysis was conducted in shallow areas, splash water, pools, and riverbanks, whereas metabarcoding analysis used surface water to prevent contamination with PCR inhibitors. Therefore, matching sampling sites may increase accuracy. This is supported by evidence that sensitivity increased when quantitative data alone were used. In addition, TFSs were conducted on the same day as water sampling, but TFSs was conducted less frequently than water sampling in this study. If TFS were performed on all water sampling, higher sensitivity could be obtained. If sensitivity is sufficiently improved by these measures, a stable and high level of positive predictivity may be obtained in arthropod eRNA as well as fish eRNA.

Cristescu^20^ discussed the possibility that eRNA enables ecological surveys with high positive predictivity (low false-positive rate) and Miyata et al.^23^ indicated that eRNA contributes to reducing false positives in river ecological surveys. The positive predictivity of fish eRNA was higher than that of fish eDNA, and the sensitivity of eRNA was higher than that of eDNA when the results of several analyses were combined. In this study, positive predictivity and sensitivity were equivalent for the eDNA and eRNA of algae. However, the positive predictivity of eRNA was higher than that of eDNA in all surveys and merged data when an expanded database was used in arthropods. Although this result is consistent with that of previous fish surveys, the sensitivity of eRNA in the merged data was lower than that of eDNA. In addition, the differences between the sensitivities of eDNA and eRNA in the merged data were smaller than those in the individual surveys. Thus, expanding the sequence database and developing high efficiency eDNA/RNA collection methods may contribute to the recovery of eRNA metabarcoding accuracy in the future.

In a previous study investigating positive predictivity, fish species that were detected only in the eDNA metabarcoding analysis were sea and brackish water fish that did not inhabit the sampling points^23^. Similarly, although saltwater algae were equally detected in the eDNA and eRNA, terrestrial arthropods tended to be harder to detect using eRNA than when using eDNA. Interestingly, the eRNA abundances of two species (*Pteronemobius fascipes* and *Nysius plebeius*) were remarkably higher than their eDNA abundances. *Pteronemobius fascipes* are found in gravel areas near riverbanks in Japan^50^ and tend to flow into rivers. There are cases where RNA abundances are higher than the DNA abundance in the body and organisms^51,52^. This suggests that if organisms, which are the source of pollution, flow into a location alive or shortly after death, they may have higher eRNA values in comparison with eDNA values. Thus, the ratio of eDNA to eRNA could be a useful indicator of false-positive results.

This study did not focus on the differences in target sequencing for metabarcoding. Several universal primers have been reported for different gene sites, especially in macro-invertebrates. At the beginning of DNA barcoding analysis, cytochrome c oxidase subunit I (COI) was used^53,54^. COI eDNA metabarcoding analysis on arthropods has been conducted for a long time, allowing the development of some universal primers. Primers for other mitochondrial regions (12S and 16S rRNA) have also been applied^27,55^. Although amounts of eRNA might vary among organisms, mitochondrial rRNA might be more abundant and stably expressed than mRNA^56^. Further studies on the lifespan and abundance of RNA among species in vivo would be important to confirm the value of eRNA analysis. Presently, the number of registered COI sequences is much higher than those for 12S rRNA and 16S rRNA. Hence, arranging universal primers may improve detection efficiency by using different genomic positions in COI^57^ or a combination of PCR primers^58^. However, comparison studies^59,60^ and a simulation^61^ indicated that 16S and 12S could be superior to COI for identifying several taxa of insects. Therefore, 12S and 16S rRNA may enable precise identification in the future by expanding the sequence database. In this study, gInsect primers (16S rRNA) could detect all Odonata species at the sampling points (Supplementary Table S1), which might be difficult to detect using COI metabarcoding.

For the environmental assessment of rivers, benthic macro-invertebrates and algae have been used as variable measures. We found that ASPT and EPT (%) values derived from the arthropods metabarcoding and saprobic score derived from the algae metabarcoding were similar to those derived from TFS. Furthermore, ASPT scores in this study were similar to the values of the survey by Tochigi prefecture in 2017 and 2020 (ASPT: 7.6–7.8 at Nakagawa-oohashi and Shin-nakabashi)^62^. Currently, eRNA analysis is less sensitive than eDNA analysis; however, merged eDNA/eRNA data can be used to estimate water quality values from TFSs. Similarly, Emilson et al.^63^ reported that DNA metabarcoding and morphologically derived macroinvertebrate metrics were positively correlated. Uchida et al.^64^ reported that the EPT index derived from eDNA showed a more positive relationship with the total nitrogen concentration than the indices calculated using the Surber net survey data. More adequate scores of species for the metabarcoding analysis could thus be determined to help assess the water environment. However, since the water quality is typically assessed using information on richness, the features of DNA/eRNA metabarcoding analysis, which are useful for comprehensive species detection, are suitable for deriving the indices. Thus, current knowledge and measures could be used in eDNA/eRNA metabarcoding analysis to improve the limitations of ecological survey using morphological evaluations^65^.

## Conclusions

To our knowledge, this study is the first to conduct a comparative eDNA/eRNA metabarcoding analysis on algae and arthropods to evaluate the performance of ecological surveys in rivers. The results indicated that both eDNA and eRNA detected a variety of species. The species composition in these analyses was remarkably different from that in the TFS, and the sensitivities were low. However, for arthropods the low sensitivity could be improved by using an expanded genomic database. Additionally, although TFSs focus on attached algae, metabarcoding analysis can detect both planktonic and attached algae; however, these differences require further investigation. For arthropods, although the sensitivity of eRNA was found to be lower than that of the eDNA, the positive predictability of eRNA was higher. This indicates that eRNA could be useful for determining false positives in eDNA analysis. Moreover, the balance between eDNA and eRNA can vary depending on the habitat of an organism. Further extensive investigations are thus required to determine the value of implementing eRNA analysis in ecological surveys.

Thus, our conclusions to the hypothesis are as follows: (1) sufficient amount of eRNA was available for metabarcoding analysis of river arthropods and algae; (2) recently-obtained arthropod nucleic acid sequences drastically improved the performance (especially, positive predictivity) of the ecological survey; (3) eRNA indicated higher positive predictivity and discriminated some unique terrestrial organisms in arthropods, but no difference between eDNA and eRNA was found in algae; (4) both eDNA and eRNA could predict water quality indices that were derived from TFS; (5) although performance should be further improved, this study suggests the potential usefulness of assessing comprehensive river ecosystem. Based on the above, eRNA metabarcoding analysis could be used to evaluate biodiversity and water quality and to provide different insights with eDNA in ecological surveys.

## Supplementary Information

Supplementary Figure S1.

Supplementary Table S1.

Supplementary Table S2.

Supplementary Table S3.

## Data Availability

The sequence data in MBIJ that support the findings of this study belongs to a third party (Bioengineering Lab. Co., Ltd., Plantbio Co., Ltd., Kanagawa Environmental Research Center and Takumi Shimizu (Keio University)). Thus, availability restrictions apply to the data used under license for the current study, and consequently, these are not publicly available. However, species data are listed in Supplementary Table S1. If you need additional information from the party, please contact the form in Bioengineering Lab. Co., Ltd. (https://gikenbio.com/contacts/) or e-mail address (dna@gikenbio.com).
