# Supplementary material for: Comparative environmental RNA and DNA metabarcoding analysis of river algae and arthropods for ecological surveys and water quality assessment
Source: Sci Rep. 2022 Nov 18;12:19828. doi: 10.1038/s41598-022-23888-1 (PMC9674700; doi:10.1038/s41598-022-23888-1)
Supplement: Supplementary file 1 — Supplementary Figure S1. [file 41598_2022_23888_MOESM1_ESM.docx]

Supplementary Figure S1. Comparison of eDNA/eRNA metabarcoding analysis performances for ecological survey in arthropods between TFS databases used.


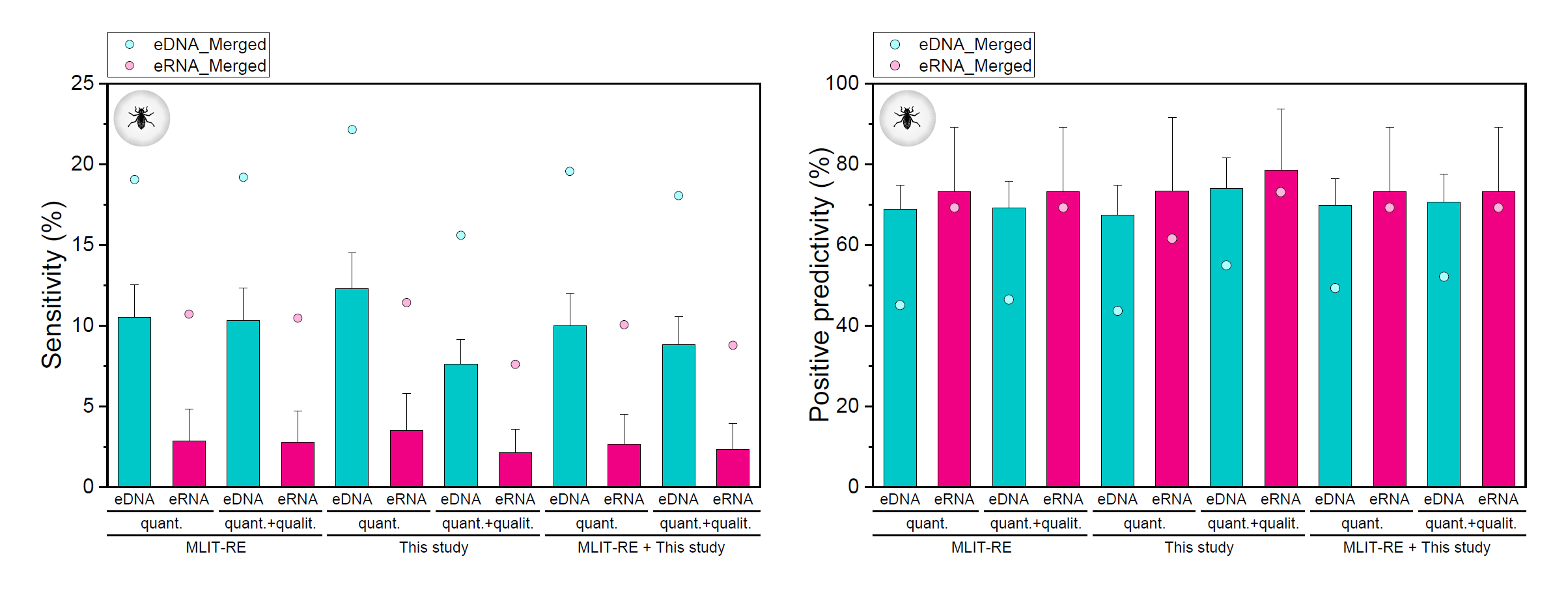


Sensitivity and positive predictivity of metabarcoding analysis in arthropods were analyzed using two different type of TFS data sets (MLIT-RE, the present study, and their integrated database). MLIT-RE: an external database (URL: http://www.nilim.go.jp/lab/fbg/ksnkankyo/ (accessed 22.5.21)). eDNA/eRNA: eDNA/eRNA metabarcoding analysis, quant.: quantitative analysis, qualit.: qualitative analysis. Dots indicates performances when individual data were merged.
